# Image Cytometric Analysis of Algal Spores for Evaluation of Antifouling Activities of Biocidal Agents

**DOI:** 10.1038/s41598-017-07362-x

**Published:** 2017-07-31

**Authors:** Bon Il Koo, Yun-Soo Lee, Mintae Seo, Hyung Seok Choi, Geok Leng Seah, Taegu Nam, Yoon Sung Nam

**Affiliations:** 1 Department of Materials Science and Engineering, Korea Advanced Institute of Science and Technology, 291 Daehak-ro, Yuseong-gu, Daejeon, 34141 Republic of Korea; 2 Marine and Heavy Duty Coatings R&D Team, KCC Central Research Institute, 17-3 Mabuk-ro 240beon-gil, Giheung-gu, 16891 Yongin-si, Gyeonggi-do Republic of Korea; 3 KAIST Institute for the NanoCentury, Korea Advanced Institute of Science and Technology, 291 Daehak-ro, Yuseong-gu, Daejeon, 34141 Republic of Korea

## Abstract

Chemical biocides have been widely used as marine antifouling agents, but their environmental toxicity impose regulatory restriction on their use. Although various surrogate antifouling biocides have been introduced, their comparative effectiveness has not been well investigated partly due to the difficulty of quantitative evaluation of their antifouling activity. Here we report an image cytometric method to quantitatively analyze the antifouling activities of seven commercial biocides using *Ulva prolifera* as a target organism, which is known to be a dominant marine species causing soft fouling. The number of spores settled on a substrate is determined through image analysis using the intrinsic fluorescence of chlorophylls in the spores. Pre-determined sets of size and shape of spores allow for the precise determination of the number of settled spores. The effects of biocide concentration and combination of different biocides on the spore settlement are examined. No significant morphological changes of *Ulva* spores are observed, but the amount of adhesive pad materials is appreciably decreased in the presence of biocides. It is revealed that the growth rate of *Ulva* is not directly correlated with the antifouling activities against the settlement of *Ulva* spores. This work suggests that image cytometric analysis is a very convenient, fast-processable method to directly analyze the antifouling effects of biocides and coating materials.

## Introduction

Marine biofouling is the undesirable accumulation of marine organisms on the surface of structures in contact with seawater such as ship hulls, propellers, and bow thrusters. The biofouling process begins by the formation of a conditioning layer, comprising organic molecules, such as polysaccharides, proteins, lipids, and proteoglycan^1–3^. The conditioning layer provides a sticky surface for the primary colonization of microfoulers (e.g., bacteria, yeasts, and diatoms) within a protective biofilm in a day. Algal spores, barnacle cyprids, and marine fungi settle as secondary colonizers in about one week of immersion under favorable environmental conditions. The adhesive molecules and the roughness of irregular colonies could attract invertebrate larvae (e.g., mussels, bryozoa, tubeworms, etc.) to the surface, which is regarded as the last stage of the marine biofouling process. The complete formation of a complex biofouling community usually takes 2–3 weeks post immersion during the spawning season^1^. These macrofoulers greatly increase the surface roughness which causes drag resistance of a ship, increasing the fuel consumption by 40–50%^4, 5^ and generating an annual cost of over USD 200 million^6^. To eliminate the settled microorganisms, dry-docking and diver cleaning are usually required, which impose significant additional costs^6^.

Various antifouling approaches have been developed. The adhesion of microorganisms is generally driven by a combination of electrostatic interaction, Van der Waals interaction, and surface morphology. Thus, the modification of parameters such as surface charge, charge density, topography, and wettability is important in controlling the settlement of marine biofoulers. For example, fouling organisms such as bacteria and *Ulva* spores prefer hydrophilic surfaces to hydrophobic ones, which motivated the development of hydrophobic coatings for antifouling applications^7^. Antifouling polymer materials, including fluorinated polymers, poly(dimethylsiloxane), and poly(ethylene glycol), have been paid much attention because they are environmentally benign^8^; however, their insufficient antifouling activity, narrow ranges of target species, and high cost restrict wide applications. Incorporation of toxic biocides to antifouling paints is the most common method to prevent the formation of the colonizing communities. Self-polishing copolymer coating technology was developed to increase the biocide portion in the copolymer and prolong antifouling effects^1, 3^. The major issue of chemical biocides is their environmental toxicity, which will be discussed in more detail. Biological enzymes have also been employed to degrade adhesive materials used for settlement due to their high environmental compatibility^9^. For instance, proteases, which hydrolyze peptide bonds at different sites, can degrade mucilage and hence prevent biofouling. However, the practical use of enzymes for antifouling applications is limited due to their high cost and relatively low antifouling activities.

Chemical biocides have been most widely used for antifouling paints because they are cost effective and exhibit high antifouling activities. Following World War II, tributyltin (TBT) was developed as an effective antifouling biocide in self-polishing paint formulations, which extended the period between dry-dockings for repainting up to five years^1, 10, 11^. In addition, TBT does not cause galvanic corrosion to steel or aluminum hulls^12^. However, TBT caused serious non-specific toxicity to various marine organisms through its effects on the mitochondrial oligomycin-sensitive Mg-ATPase^13, 14^. As a well-known example, in the Arcachon Bay in France, TBT caused an estimated loss of USD 147 million through the reduced production of oysters. More serious and pervasive development of male sexual organs in female marine organisms was also discovered. In 2001, the International Maritime Organization (IMO) banned the application of TBT-based paints by 2003 and the presence of the paints on ship surfaces by 2008^7, 11^. This action stimulated the development of tin-free surrogate biocides for antifouling paints. However, the antifouling effects of emerging tin-free agents in antifouling paint formulations are not fully understood, though multiple biocides are generally used in combination for a commercial product. It is generally recognized that the antifouling efficiency of the mixed biocides depends on the type of ships, operating conditions, and seawater^1^. Thus, to optimize the antifouling performance, it is important to figure out the precise antifouling effects of each biocide in antifouling paint formulations against the target species.

Until now, most studies on surrogate biocides have focused on their toxicity^15, 16^. However, it is not clear whether the toxicity of biocides can be directly correlated with their antifouling activities. To design new paint formulations of antifouling paints, it is critically important to directly evaluate the antifouling activities of biocides in a quantitative manner. The efficacy evaluation of antifouling paint products has been mostly conducted through panel testing on static floating rafts^1^. This field test is very expensive, time-consuming, and susceptible to environmental changes. Moreover, the antifouling activity of individual biocides and mixed formulations against specific target foulers cannot be clearly determined. Therefore, it is very important to develop a new methodology for the quantitative assessment of antifouling efficacy under a well-controlled environment in a laboratory.

In this study, we suggest a simple method to quantitatively analyze the antifouling activity of biocides against marine green alga, *Ulva prolifera*, which is a dominant species causing soft marine fouling problem. Motile spores of *Ulva* can swim using flagella to actively seek surfaces appropriate for stable settlement^8^. Biocides used in our work are bis-{1-hydroxy-2[*H*]-pyridine thionate-*O,S*}-Cu (copper pyrithione, or CuPT), bis-{1-hydroxy-2[*H*]-pyridine thionate-*O,S*}-Zn (zinc pyrithione, or ZnPT), *N*-dichlorofluoromethylthio-*N*′*,N*′-dimethyl-N-*p*-tolyl-sulphamide (Preventol A5S, or P-A5S), 3-(3,4-dichlorophenyl)-1,1-dimethylurea (Preventol A6, or P-A6), zinc ethylene-bis-dithiocarbamate (Zineb), 4,5-dichloro-2-*n*-octyl-4-isothiazolin-6 (Sea-Nine 211 N, or S-211N), and 2-(*p*-chlorophenyl)-3-cyano-4-bromo-5-trifluoromethyl pyrrole (Econea, or EC). Our method to evaluate the antifouling efficacy of the biocides is based on image cytometric analysis^17^. Image analysis was carried out using the intrinsic fluorescence of chlorophylls in the spores settled on a glass substrate. The threshold value of fluorescence intensity, and the size and shape of spores were determined and then used for image cytometric analysis. The cell counting procedures are very fast and reproducible enough to evaluate the antifouling activities of biocides under various conditions, including the kind of biocides, concentrations, and combination of different biocides. We also examined the effects of biocides on the morphology of *Ulva* spores, the amount of adhesive pad materials, and the growth rate of *Ulva*, and discussed their correlation with the antifouling effects of biocides.

## Results and Discussion

### Image Cytometric Analysis

**Figure 1:**
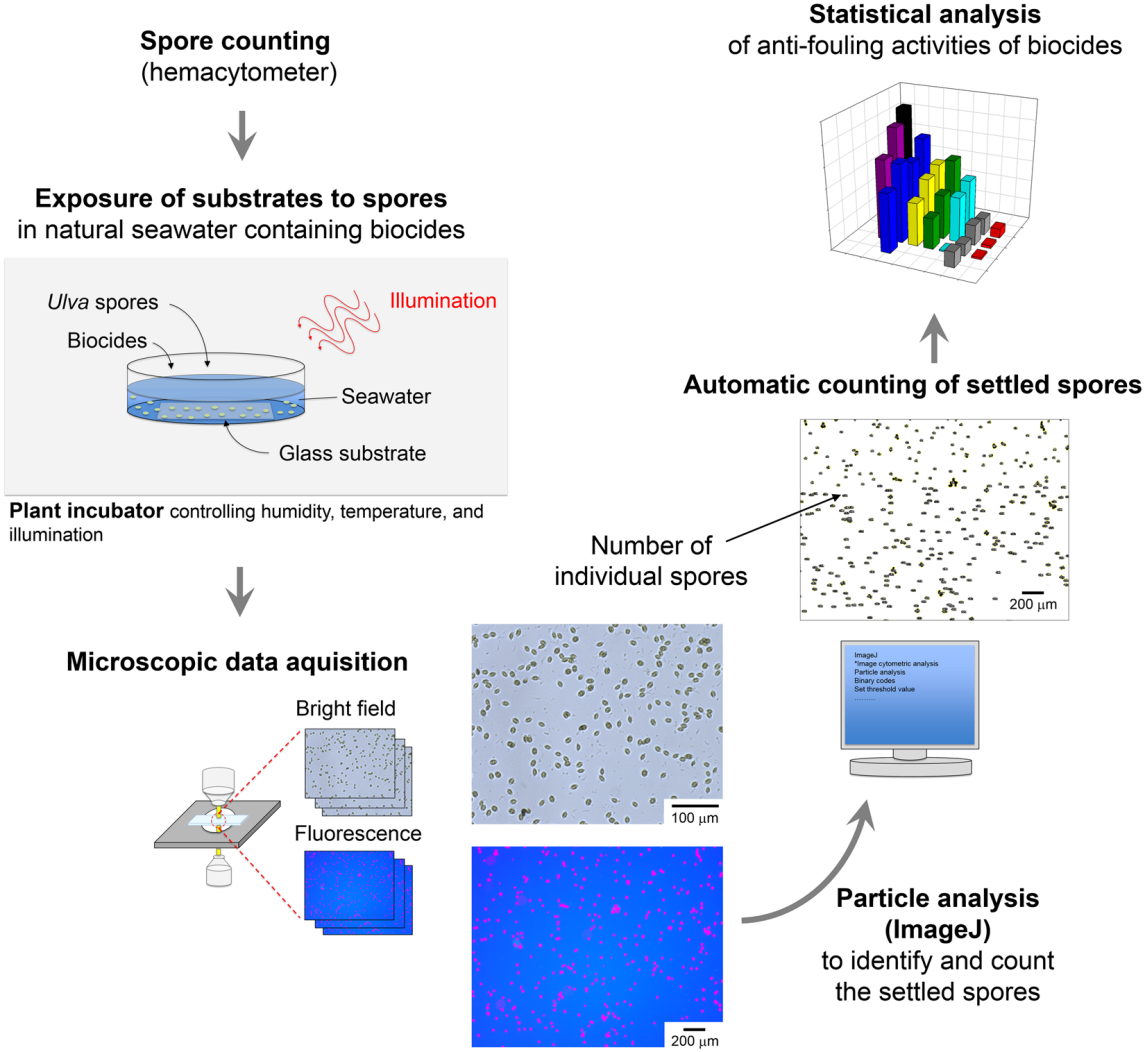
Schematic illustration of image cytometric analysis to quantitatively determine the antifouling activity of biocides.

To quantitatively determine the antifouling effects of biocides, we devised a new method based on image cytometric analysis using intrinsic fluorescence of chlorophylls in the photosynthetic *Ulva* spores, as schematically described in Fig. 1. The absorption spectrum of chlorophyll exhibits strong peaks at 430 nm and 660 nm, and its fluorescence emission has a peak at 685 nm^18^. *Ulva* spores were incubated for 24 h under the summer condition in the presence of a cover glass, and then the fluorescence images of the spores settled on the surface of the cover glass were taken on a fluorescence microscope. The fluorescence image was converted into black and white images using ImageJ, and then each black dot (the settled spore) was assigned a number. The number and area of the settled spores were calculated using the particle analysis function. The number of spores determined by image analysis matched the results obtained from a manual counting of several hundred spores with an error range of <10%, confirming the preciseness of the image cytometric analysis. In some cases, the image analysis counted clustered spores as one spore having a larger area, which led to the underestimation of the number of spores. To increase the preciseness of image cytometry, the sum of the total area of the settled spores was divided by the average area of a single spore (5.0 × 10^3^ μm^2^), so such underestimation of the spore number was minimized. The image processing procedures to obtain the spore areas using ImageJ are described in detail in the Supporting Information. Using this approach, 15–20 fluorescence images (2.4 mm × 1.8 mm) were analyzed to precisely determine the average number of the settled spores within an error range of <3%. The efficiency of the spore settlement was defined as the ratio of the number of the settled spores to the initial number of spores added to the medium.

### Effects of Biocides on Spore Settlement

**Figure 2:**
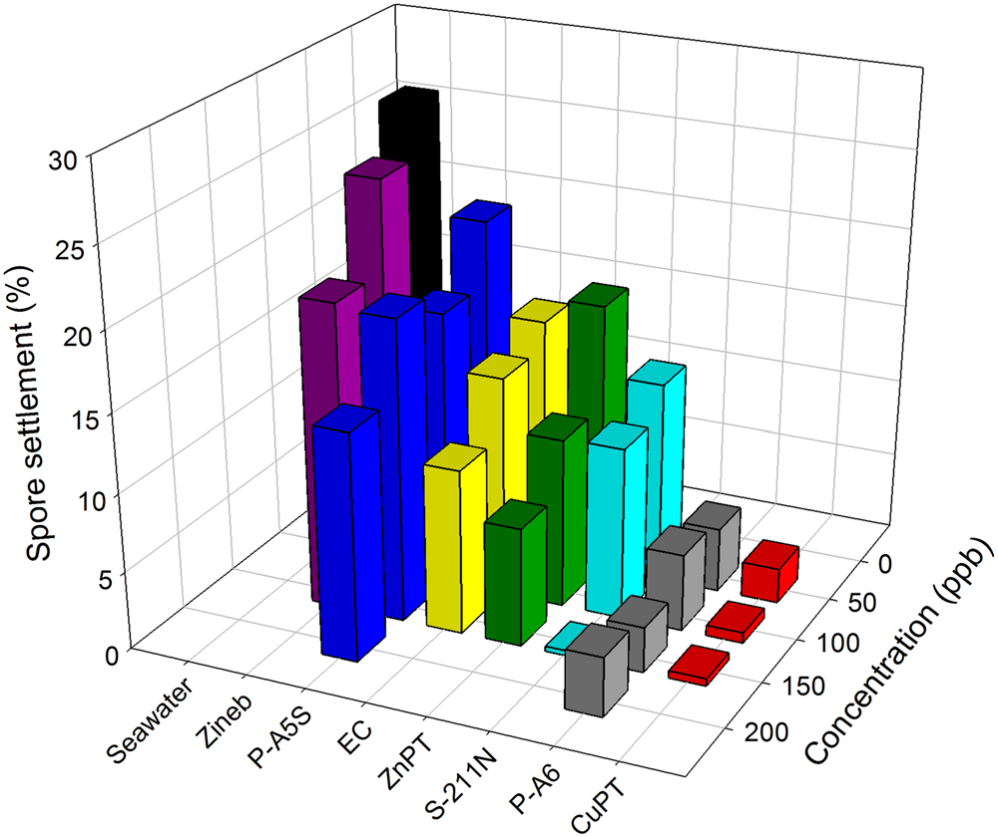
Settlement efficiency of *Ulva* spores in natural seawater containing each biocide as a function of biocide concentration (n = 3). The values and standard deviations are presented in Supplementary Table 1.

The effects of seven commercial biocides, ZnPT, CuPT, P-A5S, P-A6, Zineb, S-211N, and EC, used for antifouling paints on the settlement efficiency of *Ulva* spores, were examined up to a concentration of 200 ppb. The settlement efficiencies of spores were plotted as a function of biocide concentrations (Fig. 2). In the absence of biocides, *Ulva* spores exhibited an average settlement efficiency of 26.01% in natural seawater after 24 h incubation. The most efficient biocide was CuPT, which exhibited settlement efficiencies of only 0.47% and 2.24% at 150 ppb and 50 ppb, respectively. P-A6, which is an organic biocide widely used to prevent the growth of sea weeds in a fish tank, also exhibited relatively low spore settlement efficiencies: 2.91% and 4.12% at 150 ppb and 50 ppb, respectively. S-211N had a significantly low settlement efficiency, 0.32% at 150 ppb, but its antifouling effect was dramatically reduced at lower biocide concentrations. Zineb and P-A5S exhibited very low antifouling activities in the concentration range examined in this work. It is known that both ZnPT and CuPT are rapidly emerging biocides as alternative compounds to TBT^19^. In particular, ZnPT has been widely used as bactericides and fungicides in a variety of commercial products, including antidandruff shampoos, adhesives, sealants, and coatings^20^. Although ZnPT and CuPT have the same ligand structure, our results indicate that their antifouling effects are very much different from each other, and the metal ion coordinated with the organic ligand is critically important for their antifouling activity for *Ulva* spores.

### Effects of Combination of Multiple Biocides on Spore Settlement

**Figure 3:**
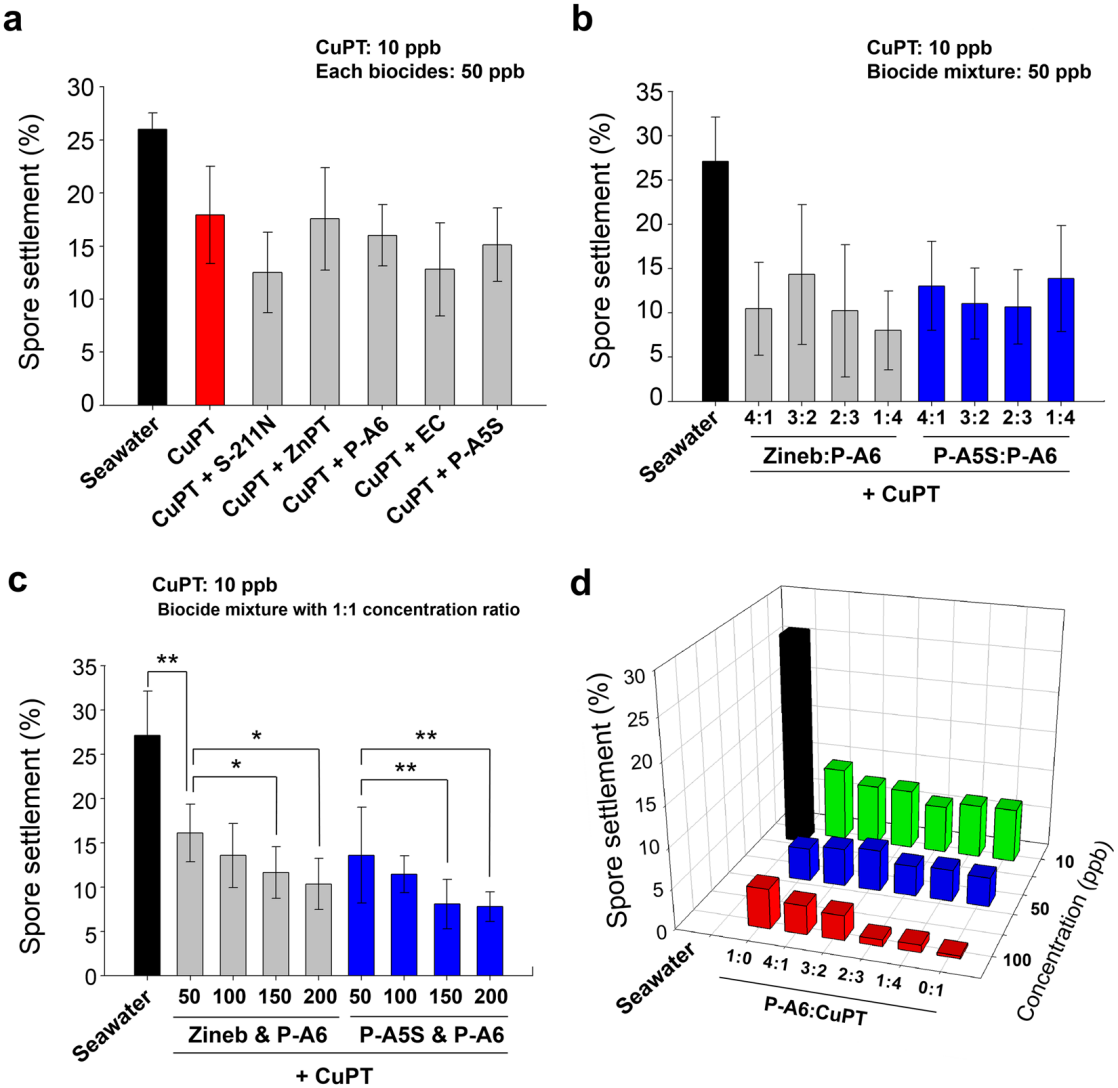
Settlement efficiency of *Ulva* spores in natural seawater containing a mixture of biocides. All of the *p*-values were < 0.001 between natural seawater and biocide solutions, while no significant difference was found between different biocides in (**a** and **b**) with n = 15. Some *p*-values below 0.01 (*) and 0.001 (**) with n = 15 are represented in (**c**). The error bars (standard deviations) and *p*-values in (**d**) are shown in Supplementary Figures 2–4 (n = 18).

In the commercial formulations of antifouling paints, a mixture of multiple biocides are generally used with a presumption that the combination of different biocides may boost and broaden the antifouling efficiency against a broader spectrum of marine biofoulers^21, 22^. Moreover, the strategy based on the combination of multiple biocides to achieve synergistic antifouling effects is reasonable because biocides adopt different modes of action for antifouling effects^23^. In this work, the fast-processable image cytometric analysis was applied to examine the antifouling effects of a large number of mixed biocides on *Ulva* spores. Among the tested biocides, CuPT was selected as a default component to examine the effect of its combination with other secondary biocides on antifouling activity because CuPT exhibited the highest antifouling effects (Fig. 2). The combination of CuPT and ZnPT was not examined because ZnPT can be transchelated to CuPT, which makes the interpretation of experimental data more complicated^24^. The concentration of CuPT was maintained at 10 ppb, at which the spore settlement efficiency was 17.93%. The final concentration of the other biocides was maintained at 50 ppb. Unfortunately, there was no significant effect of the added secondary biocides on the spore settlement efficiency (Fig. 3a). The mixtures of biocides with various ratios were also used to examine the effects of secondary biocides added to the 10 ppb CuPT. In this case, the mixtures of P-A6 + Zineb and P-A6 + P-A5S were examined as the secondary biocides at the same concentration of 50 ppb. Unfortunately, no significant difference was observed with different mixture ratios of the secondary biocides (Fig. 3b). Although the number of settled spores was not affected by the composition of the secondary biocides, the antifouling effect was increased for both of the secondary biocides as the concentration of the biocide mixtures was increased (Fig. 3c). Lastly, the antifouling effects of the mixtures of CuPT and P-A6 with various ratios and concentrations were examined (Fig. 3d). There was no significant variation in the mixture of P-A6 and CuPT at different ratios of biocides, though at 100 ppb the antifouling activity was increased with the increased amount of CuPT compared to P-A6. The spore settlement efficiencies at various ratios of P-A6 to CuPT, shown in Fig. 3d, are presented again at three different concentrations with error bars in Supplementary Figures 2–4.

The effect of CuPT seems to mainly determine the overall antifouling activity of the biocide mixture at 100 ppb. The dependency of the activity on the biocide concentration was clear while the effect of biocide combination was not appreciable. These results indicate that the tested biocides do not exhibit a significant synergistic biocidal effect in the antifouling activity against the settlement of *Ulva* spores. Although limited only to the marine biofouling against *Ulva prolifera*, our study suggests that the adjustment of the biocide concentration at the surface of antifouling paints through the controlled release of biocides can be critically important, while the combination effect of different biocides in the paint formulations is not clear.

However, the synergistic effects were expected due to the existence of multiple biocidal modes of action, as discussed above. The pyrithione complexes are known to disrupt the integrity of cell membranes and pH gradients, reduce intracellular ATP levels, and complex with metal ions in the cell^25–27^. S-211N is an electrophile that can strongly react with the nucleophile cysteine in proteins and peptides, inhibiting the algal cell division^28, 29^. P-A6 can specifically inhibit photosystem II^29^. Note that our results are limited to *Ulva*, and further analysis is still underway to discover the experimental parameters to enhance the antifouling effects through the combination of different biocides. Synergistic effect will be highly beneficial in decreasing the environmental problems caused by biocides. In particular, synergistic combination will allow for reducing the total amount of toxic biocides released to the environment while obtaining the same level of antifouling activity.

### Spore Morphology and Adhesive Pads

**Figure 4:**
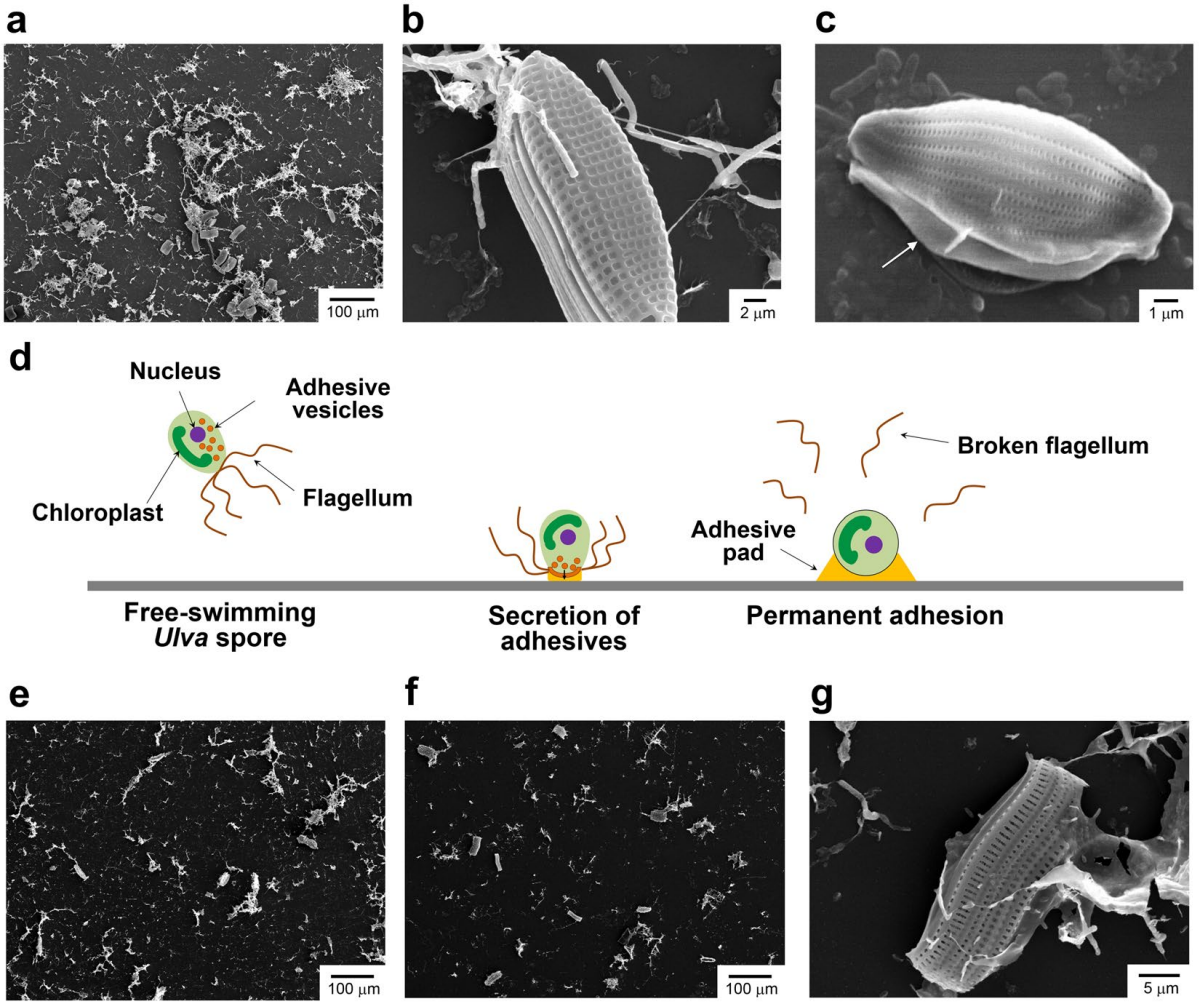
(**a**–**c**) SEM images of the surface of glass exposed to *Ulva* spore suspension in natural seawater without biocides under the summer condition for 24 h. (**d**) Schematic description of settlement process of *Ulva* spores. SEM images of the surface of glass exposed to *Ulva* spore suspension in natural seawater containing CuPT at concentrations of 10 ppb (**e**) and 100 ppb (**f** and **g**) under the summer condition for 24 h.

The scanning electron microscopy (SEM) image of a cover glass incubated with *Ulva* spores in fresh natural seawater under the summer condition shows the presence of spores and fibrous structures (Fig. 4a). The fibrous structures appear to be the broken flagella detached from the body of *Ulva* spores. Motile *Ulva* spores swim using flagella, which are lash-like whiskers that protrude from the spore body, to actively search for substrates for suitable settlement^8^. The spores secret a glycoprotein adhesive from cytoplasmic vesicles to attach to the surface, forming a gel matrix generating a favorable local surface chemistry for the settlement of spores^30, 31^. Higher magnification images show the ellipsoidal shape of an *Ulva* spore with a long-axis length of about 10 μm (Fig. 4b,c). Adhesive pad materials between the spore and the substrate are indicated using an arrow in Fig. 4c. Small rod-shaped microbes were also found to stick to the substrate. The settlement process of *Ulva* spores is schematically described in Fig. 4d.

Next, we examined the effects of biocides on the morphology and adhesive pad deposition of *Ulva* spores. The spores were incubated for 24 h under the summer condition in natural seawater containing 10 ppb (Fig. 4e) and 100 ppb CuPT (Fig. 4f,g), respectively. The number of spores and the amount of the adhesive pad were apparently reduced in the presence of CuPT. The residual adhesive pad materials on the substrate indicate that the spores attempted to adhere to the substrate, but they were easily detached presumably because CuPT weakened the binding affinity of the spores. The overall morphology of the spore was not appreciably changed by 100 ppb CuPT. Unbound spores were also collected to compare their morphologies. No significant differences were observed, although their surfaces were very neat without adhesive pads and microbes (Supplementary Fig. 5). The result indicates that CuPT does not affect the outer membrane of spores, and the interaction between the adhesive pad and the spore body is critically important for the stable adhesion of spores to the substrate.

### Analysis of Adhesive Pads

Fourier Transform Infrared (FTIR) spectroscopy was used to analyze the chemical functional groups of the adhesive pads. Two characteristic absorption peaks for proteins and lipids appeared at 1650 cm^−1^ and 1750 cm^−1^, corresponding to the amide I of protein and ester carbonyl stretch of lipids, respectively (Supplementary Fig. 6). The broad band around 1080 cm^−1^ can be assigned to the C-O and C-C stretch vibrations coupled to C-O-H bending from polysaccharides^32^. The FTIR band assignment indicates that the adhesive pad consists of phospholipids, proteins, and polysaccharides. It is known that the cytoplasmic vesicles secret a glycoprotein adhesive for the settlement of *Ulva* spores^30^. The phospholipid-protein ratios can be quantitatively analyzed from the following equation: moles lipid per kg protein = (*I*_1_/*I*_2_ − 0.0175)/0.2590, where *I*_1_ and *I*_2_ are the band intensity in the ranges of 1730–1800 cm^−1^ and 1650–1700 cm^−1^, respectively^33^. The result indicates that the adhesive pads of *Ulva* spores contained 11.4 moles of phospholipids per kg protein as the measured *I*_1_ and *I*_2_ were 9.79 and 3.28, respectively.

To visualize the adhesive pads, cyanine 5-*N*-hydroxysuccinimide ester (Cy5-NHS), an amine reactive dye widely used for fluorescence labeling of peptides and proteins, was used. Spores were settled on a cover glass for 24 h under the summer condition, and 1 mg mL^−1^ NHS-Cy5 was added to the settled spores, followed by incubation for 4 h. The maximum wavelengths of excitation and emission of Cy5 are about 650 nm and 670 nm, respectively. In our microscope, the Cy5-labeled adhesive pads were excited using a red light source with a maximum wavelength of 620 nm (Y5 filter, λ_ex_ = 620 ± 30 nm, λ_em_ = 700 ± 32.5 nm). At this wavelength, unfortunately, *Ulva* spores were also detected, so the particle analysis was implemented again, as described above, and the area of spores was excluded to obtain the precise area of the adhesive pad (Supplementary Fig. 7).

**Figure 5:**
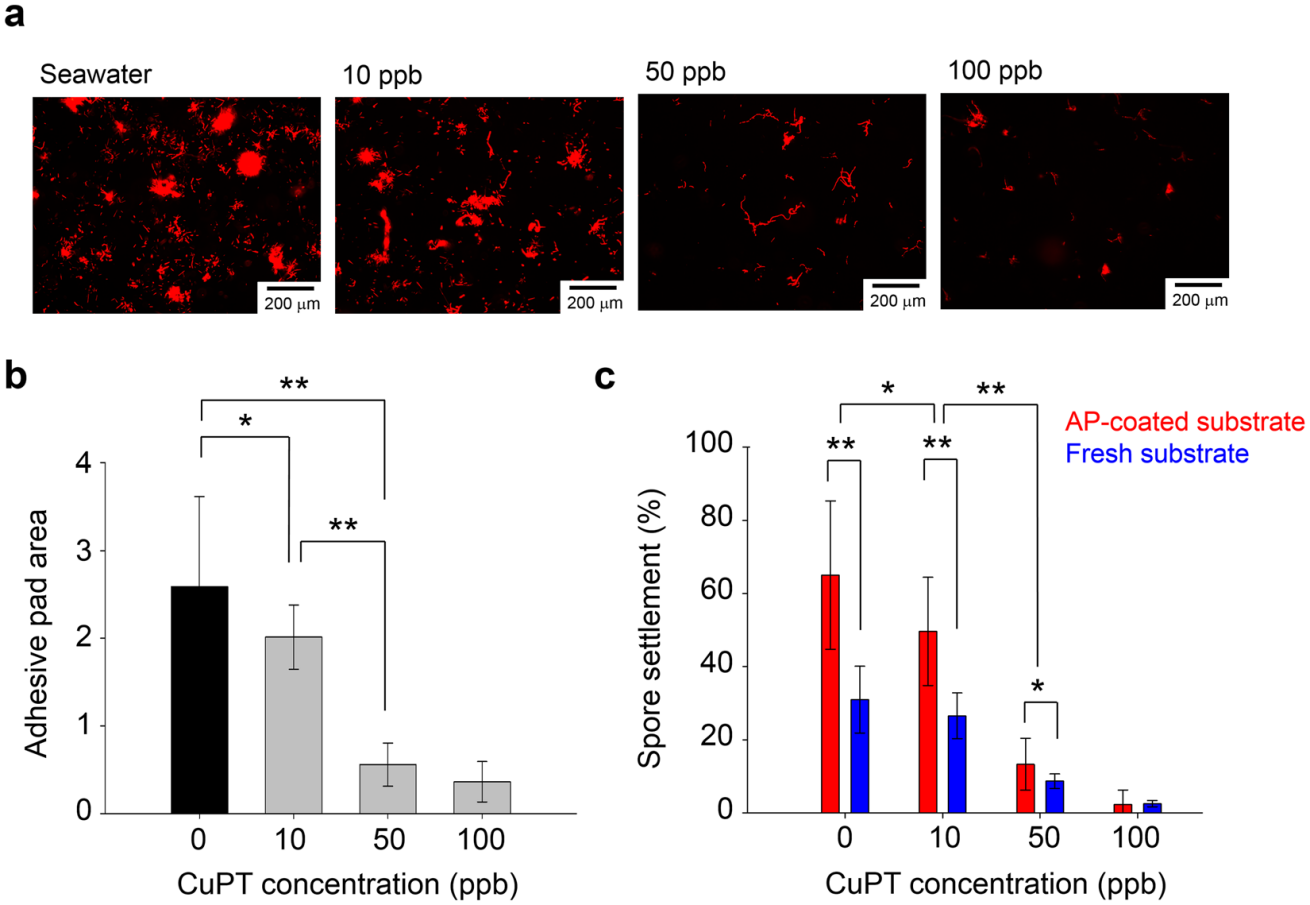
(**a**) Fluorescence microscopy images of the Cy5-treated surface of glass exposed to *Ulva* spore suspension in natural seawater containing CuPT at various concentrations under the summer condition for 24 h. (**b**) Quantitative comparison of adhesive pad areas calculated from images in (**a**). (**c**) Spore settlement efficiency on the fresh and AP-coated glass surfaces exposed to *Ulva* spore suspension in natural seawater without biocides under the summer condition for 24 h. Some *p*-values below 0.05 (*) and 0.001 (**) with n = 18 or 19 are represented in (**b** and **c**).

To determine the effects of CuPT on the deposition of the adhesive pad, three different concentrations of CuPT, 10 ppb, 50 ppb, and 100 ppb, in natural seawater were used. The fluorescence microscopy images clearly exhibit the decreased area of adhesive pad with the increased concentration of CuPT (Fig. 5a). The adhesive pad areas were quantitatively compared through image analysis using ImageJ (Fig. 5b). It is clearly shown that the amount of adhesive pads was appreciably reduced when the CuPT concentration was increased from 10 ppb to 50 ppb. This result is very well consistent with the spore settlement efficiency, as shown in Fig. 5c, which indicates that the formation of adhesive pads is very important for the stable binding of spores to the substrate.

To further demonstrate the importance of adhesive pads, we carried out the spore settlement experiment using the substrates that were already coated by adhesive pads. Briefly, the cover glass pre-coated with adhesive pads (denoted ‘AP-coated’) was prepared by exposing a fresh substrate to spores for 3 days under the summer condition. The spores were physically removed using a plastic blade, and it was confirmed that no residual spores remained under the optical microscope. Fresh and AP-coated substrates were exposed to *Ulva* spores in natural seawater and biocide solutions, and then the numbers of settled spores were calculated using the image cytometric analysis. The spore settlement efficiencies were twice higher in the AP-coated substrates compared to those in fresh substrates regardless of the biocide concentrations. This result is consistent with previous findings that the presence of a biofilm can facilitate the settlement of some algal zoospores^34^.

### Inhibition Effects of Biocides on the Growth of *Ulva*

**Figure 6:**
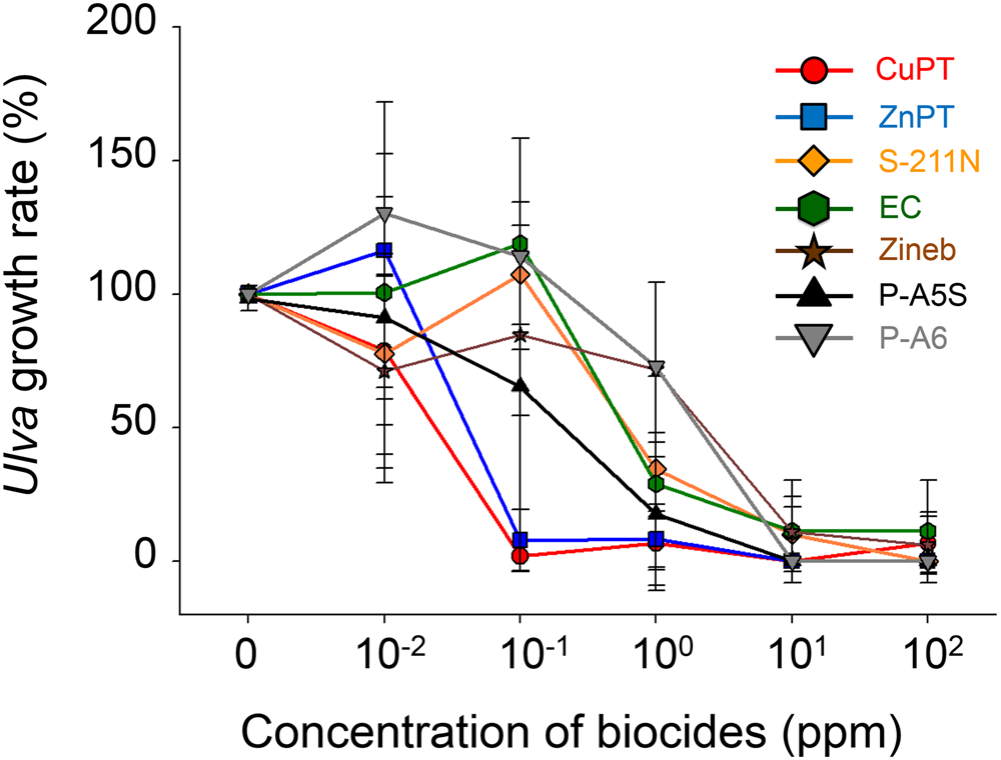
*Ulva* growth rate in natural seawater containing each biocide as a function of biocide concentration. The *Ulva* was incubated under the winter condition for one week. The error bars represent the standard deviations with n = 9.

Biocides are basically cytotoxic compounds that inhibit biologically essential functions for the survival of marine biofoulers. Although inhibiting the adhesion of spores is the most efficient way to suppress the formation of biofouling colonies on the substrate, it is also possible that biocides suppress the growth of *Ulva prolifera*. This effect can reduce the hydrodynamic drag forces caused by the growing biofoulers. Thus, to determine the effects of biocides on the growth of *Ulva prolifera*, the body of *Ulva* was cut into smaller rods of 3 cm in length and placed in 6 mL of natural seawater containing the biocides at different concentrations in a six-well plate. The length of the body was measured after 1-week incubation under the winter condition. The growth rate of *Ulva*, defined as the ratio of the growth length of *Ulva* in a biocide solution to that in natural seawater, is plotted as a function of the biocide concentration, as shown in Fig. 6. Interestingly, the two pyrithiones (CuPT and ZnPT) exhibited the most efficient suppression of *Ulva* growth in a very similar manner. There was only about 10% growth of *Ulva* even at the concentration of 10 ppb. The other biocides exhibited the similar level of growth rate. Note that ZnPT was very effective for the suppression of the growth of *Ulva*, but it exhibited a very low activity for the settlement of spores. The results indicate that the antifouling activity of biocides is not simply based on their non-specific cytotoxicity. There is no correlation between the biocide activities on spore settlement and the growth of *Ulva*, although CuPT exhibited excellent inhibition effects on both of them.

## Conclusion

This work demonstrated that image cytometric analysis is an effective means of quantitatively and quickly determining the antifouling activities of commercial biocides. *Ulva prolifera* was employed as a target organism because it is a dominant species causing marine soft fouling. In particular, the intrinsic fluorescence of chlorophylls in the *Ulva* spores allowed for the direct determination of the number of spores settled on a substrate without any labeling. When the shape and threshold intensity of spores were determined, the image analysis procedures took only a few minutes to calculate the total number of spores, which was mostly in the range of 10^2^–10^3^, in one image having a dimension of 2.4 mm × 1.8 mm. This fast-processable method enabled analysis of a large number of samples to determine the effect of biocides on the settlement of *Ulva* spores on a substrate. Our results indicate that CuPT exhibited the highest antifouling activity among seven commercial biocides, while ZnPT, which has the same ligand structure, showed a relatively low activity. However, these two pyrithiones had very similar effects on the growth inhibition of *Ulva prolifera*. When multiple biocides were used, no synergistic biocidal effects were observed in the antifouling activity against the settlement of *Ulva* spores, although multiple biocidal mechanisms imply the possible existence of synergistic effects. The effect of the concentration of biocides was obvious in the range of 10–100 ppb, indicating that the controlled release of biocides on the surface of paints is very important. No morphological changes of *Ulva* spores were found in the presence of biocides, but the amount of adhesive pads was gradually decreased with the increased concentration of biocides, and the number of spores settled on the substrate was very well correlated with the amount of adhesive pads. We expect that the image cytometric analysis can be widely used as a simple method to directly evaluate the antifouling performances of biocide formulations and antifouling materials, which can facilitate the activities in the field of marine antifouling research.

## Experimental Section

### Materials

*Ulva prolifera* was obtained from the Marine Green Algal Resources Bank at Pukyong National University (Busan, Republic of Korea). Natural seawater was collected in the shore of Seocheon in Republic of Korea. Provasoli Enriched Seawater (PES) medium was supplied from the Korea Marine Microalgae Culture Center (Busan, Republic of Korea). Cy5-NHS was purchased from GE Healthcare Life Science (Buckinghamshire, UK). Glutaraldehyde (Grade I) and dimethyl sulfoxide (DMSO) were purchased from Sigma-Aldrich (St. Louis, MO, USA). CuPT, ZnPT, P-A5S (Riedel-de Haën, Switzerland), P-A6, Zineb, S-211N (Rohm and Haas, Philadelphia, PA, USA), and EC were provided by KCC Co. (Seoul, Republic of Korea). The molecular structures of each biocides are described in the Supporting Information (Supplementary Fig. 1).

### Culture of *Ulva prolifera*

*Ulva* was cultured in enriched natural seawater. PES powder was dissolved at a concentration of 2% in 1 L of the filtered and sterilized natural seawater, which was autoclaved at 120 °C for 60 min, followed by 0.22 μm filtration in vacuum. *Ulva* was cultured in a 6-well tissue culture plate for the growth of *Ulva* and spore release in plant incubators, which were programed to control the light intensity, temperature, illumination periods, and humidity with a period of 24 h (Growth Chamber, JSPC-200C, JS Research Inc., Gongju, Republic of Korea). The summer condition for the release of *Ulva* spores was set to have a 16 h:8 h light:dark cycle with a high luminance (~4000 lux) at 20 °C, while the winter condition for the proliferation of *Ulva* was an 8 h:16 h light:dark cycle with 1000 lux at 10 °C.

### Collection of *Ulva* Spores

Under the summer condition, the spores were released from *Ulva* post 5–7 day culture. The natural seawater from a 6-well-plate was transferred to a 50 mL plastic tube and centrifuged at 1,500 rpm for 4 min (Labogene 1580 R, Seoul, Republic of Korea). The collected spores were observed under an optical microscope (Leica DMI 3000 B, Leica Microsystems, Wetzlar, Germany). The number of spores was determined using a hemocytometer.

### Spore Settlement Analysis

CuPT and ZnPT were dissolved in DMSO at a concentration of 2 mg mL^−1^, and other biocides at 20 mg mL^−1^ in the same solvent. The final concentration of biocides was adjusted by diluting the solution with fresh natural seawater. A cover glass (2.2 cm × 2.2 cm) was placed in a Petri dish with a diameter of 3.5 cm, and then the biocide solution and the spore suspension were added. After incubation for 24 h, the settled spores were observed under a fluorescence microscope.

### Electron Microscopic Analysis

The spores settled on the cover glass were immersed in 2.5% of glutaraldehyde for 30 min at room temperature, washed with deionized water five times, and then dehydrated by freeze-drying (IlshinBioBase, Dongducheon, Republic of Korea) for 12 h. The dehydrated cells were coated with platinum with a thickness of about 3 nm using a sputter coater (BAL-TEC SCD 005, Leica Biosystems, Wetzlar, Germany) and then observed using SEM (Hitachi S-4800, Tokyo, Japan).

### Determination of *Ulva* Growth Rate

Six milliliters of fresh natural seawater or the biocide solutions were added to a 6-well plate, and *Ulva* with a length of 3 cm was placed in each well and cultured for 1 week under the winter condition. The length of *Ulva* was measured using a ruler.

### Statistical Analysis

Statistical analysis was performed using a standard Student’s t-test with a minimum confidence level (p-value) of 0.01 for significant statistical difference. The sample size is denoted by ‘n’ for each case.

## Electronic supplementary material

Supplementary Information
