# Supplementary material for: Image Cytometric Analysis of Algal Spores for Evaluation of Antifouling Activities of Biocidal Agents
Source: Sci Rep. 2017 Jul 31;7:6908. doi: 10.1038/s41598-017-07362-x (PMC5537363; doi:10.1038/s41598-017-07362-x)
Supplement: Supplementary file 1 — Supplementary Information [file 41598_2017_7362_MOESM1_ESM.pdf]

Supplementary Information

Image Cytometric Analysis of Algal Spores for Evaluation of Antifouling Activities of Biocidal Agents

Bon Il Koo[1], Yun-Soo Lee[2], Mintae Seo[1], Hyung Seok Choi[1], Geok Leng Seah[1], Taegu Nam[2], and Yoon Sung Nam[1,3,]*

[1]Department of Materials Science and Engineering, Korea Advanced Institute of Science and Technology, 291 Daehak-ro, Yuseong-gu, Daejeon, 34141, Republic of Korea

[3]Marine and Heavy Duty Coatings R&D Team, KCC Central Research Institute, 85 Mabuk-dong, Giheung-gu, Yongin-si, Gyeonggi-do, 16891, Republic of Korea

[3]KAIST Institute for the NanoCentury, Korea Advanced Institute of Science and Technology, 291 Daehak-ro, Yuseong-gu, Daejeon, 34141, Republic of Korea

* To whom correspondence should be addressed.
E-mail: yoonsung@kaist.ac.kr; phone: +82-42-350-3311; fax: +82-42-350-3310

Detailed procedures to obtain spore areas from image cytometric analysis using ImageJ

Let us start with image transformation for cell counting. In the menu bar of ImageJ program, click <Image> → <Adjust> → <Threshold>.

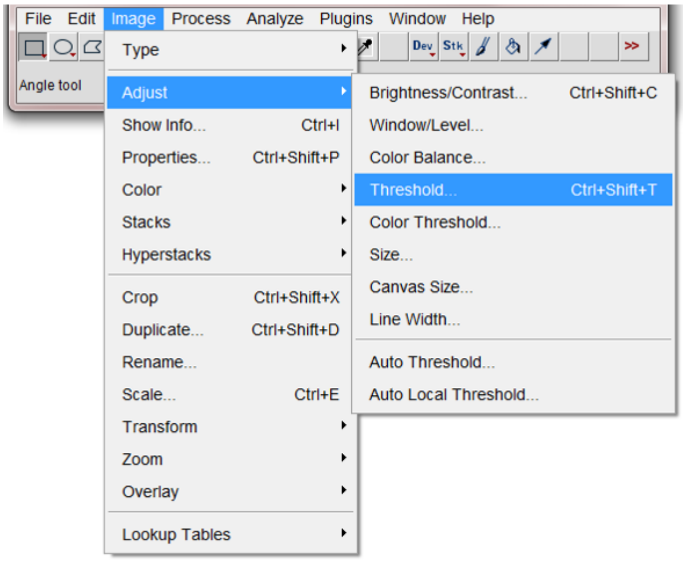

Color the background uniformly while keeping the cells to be counted as they are.

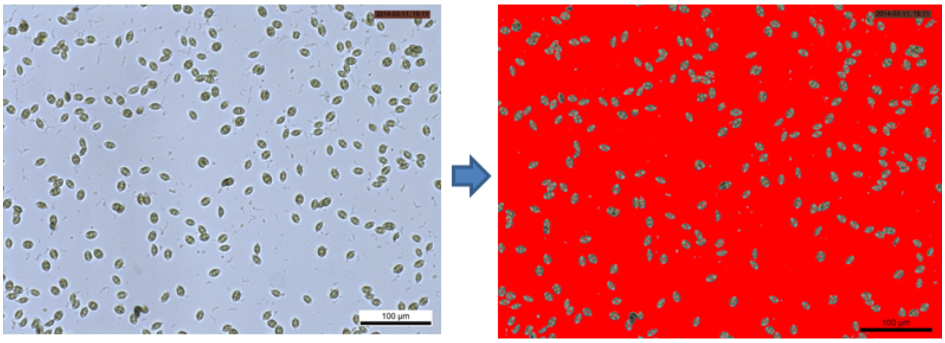

If there are overlapping cells, the number of cells can be underestimated, so adjust the hue, saturation, and brightness as much as possible so that the outer background completely surrounds the cells.

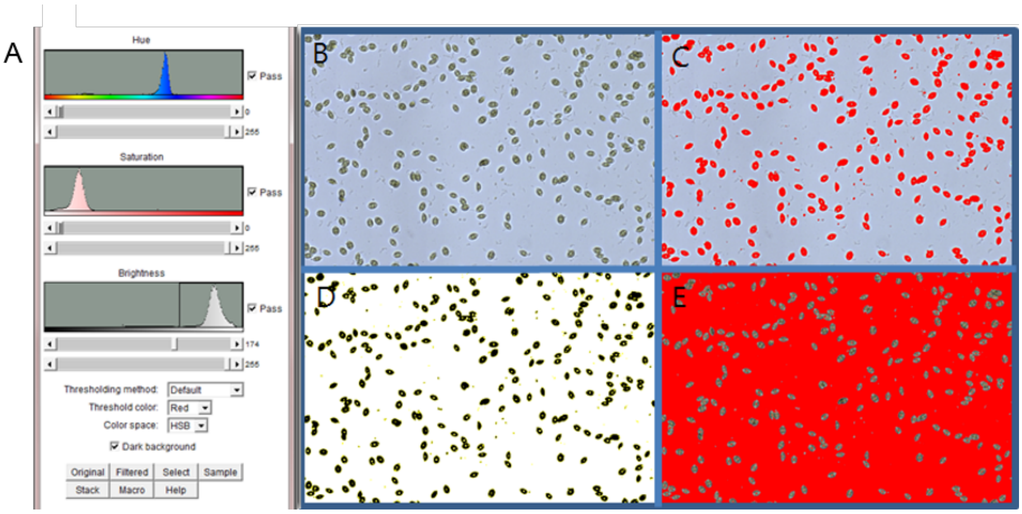

The best way to obtain an image that contains the right color is to include the whole peak area in the brightness, but only partially include the saturation as shown below.

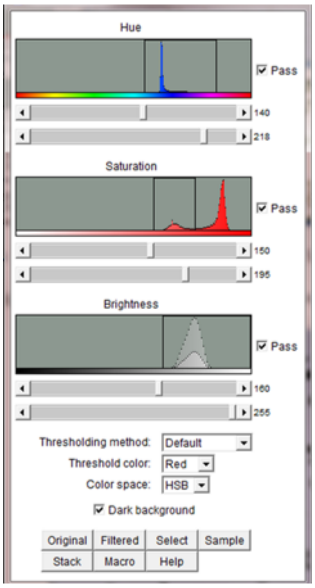

This is a typical image obtained from the image adjustment.

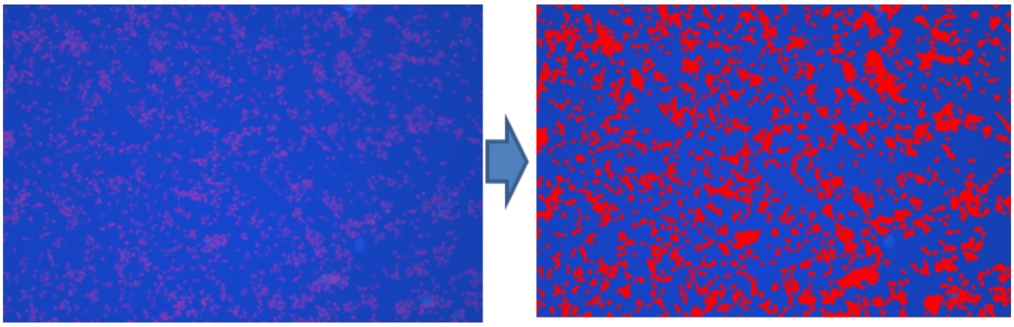

If the background is not clear, you can adjust it in <Process> → <Subtract Background>. If this function does not work properly, you can reset the <Process> → <Find Edges> to get the image with the correct boundaries.

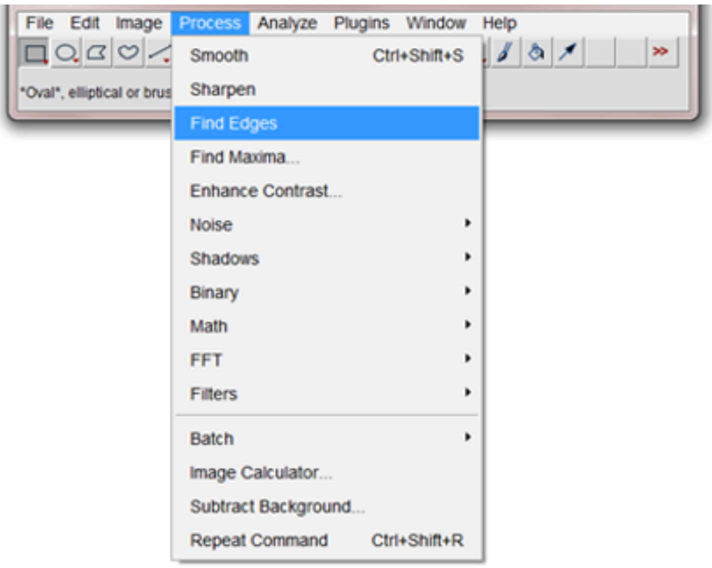
File Edit Image Process Analyze Plugins Window Help
"Oval", elliptical or brus
Smooth Ctrl+Shift+S
Sharpen
Find Edges
Find Maxima...
Enhance Contrast...
Noise
Shadows
Binary
Math
FFT
Filters
Batch
Image Calculator...
Subtract Background...
Repeat Command Ctrl+Shift+R

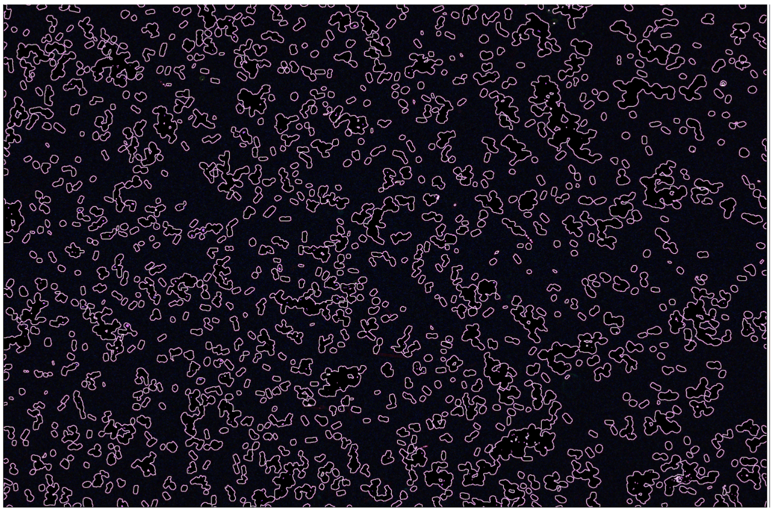

To measure the number of cells, click <Analyze> → <Analyze Particles>, then click <Display results>, <Clear results>, <Add to Manger>, then <OK>.

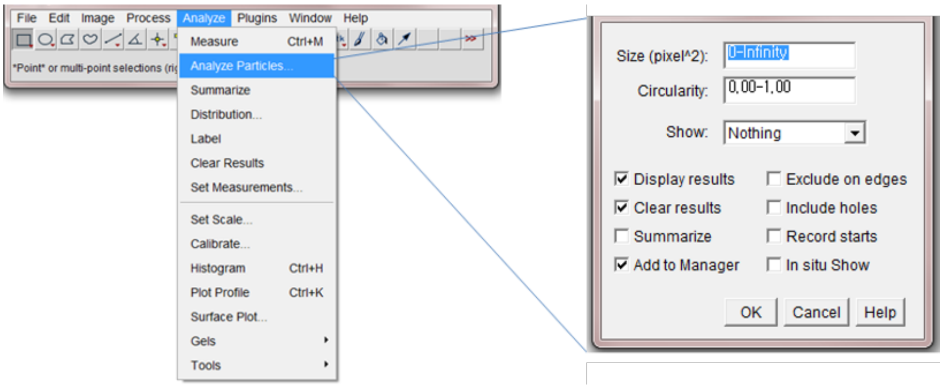

Then, you can obtain the following results.

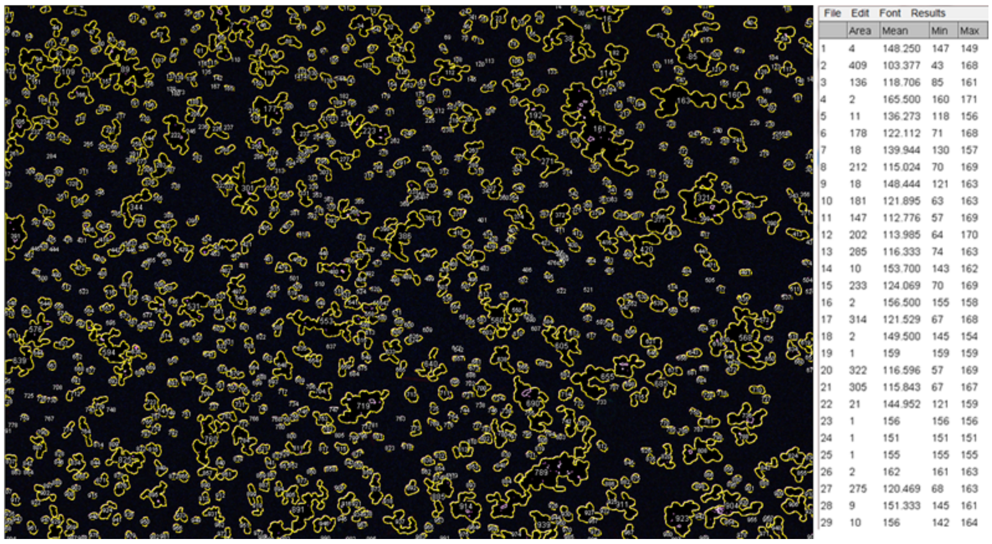

File Edit Font Results

	Area	Mean	Min	Max
1	4	148.250	147	149
2	409	103.377	43	168
3	136	118.706	85	161
4	2	165.500	160	171
5	11	136.273	118	156
6	178	122.112	71	168
7	18	139.944	130	157
8	212	115.024	70	169
9	18	148.444	121	163
10	181	121.895	63	163
11	147	112.776	57	169
12	202	113.985	64	170
13	285	116.333	74	163
14	10	153.700	143	162
15	233	124.069	70	169
16	2	156.500	155	158
17	314	121.529	67	168
18	2	149.500	145	154
19	1	159	159	159
20	322	116.596	57	169
21	305	115.843	67	167
22	21	144.952	121	159
23	1	156	156	156
24	1	151	151	151
25	1	155	155	155
26	2	162	161	163
27	275	120.469	68	163
28	9	151.333	145	161
29	10	156	142	164

The counted number, <Area>, <Mean>, <Min>, and <Max> are displayed. Store only the <Area> in a MS-Excel sheet.

Name	Structure
Copper Prythione (CuPT)	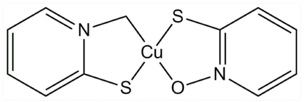
Zinc Prythione (ZnPT)	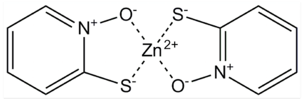
Preventol A5S (P-A5S)	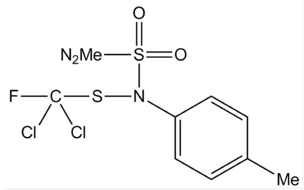
Preventol A6 (P-A6)	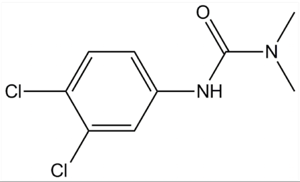
Zineb	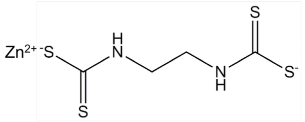
Sea-Nine 211N (S-211N)	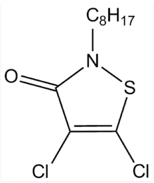
Econea (EC)	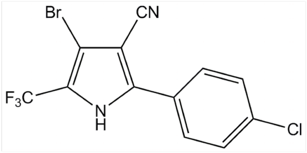

Figure S1. Molecular structures of biocides used in this study.

Table S1. Settlement efficiency of Ulva spores in natural seawater containing particular biocides as a function of biocide concentration with value and standard deviation.

Concentration (ppb)		0	50	100	150	200
Settlement efficiency (%)	**Seawater**	26.0 ± 1.5	-	-	-	-
	**Zineb**	-	25.2 ± 6.2	19.6 ± 6.4	-	-
	**P-A5S**	-	21.4 ± 4.5	17.5 ± 5.6	19.2 ± 7.3	15.3 ± 1.0
	**EC**	-	15.6 ± 0.1	14.1 ± 6.9	10.6 ± 1.9	-
	**ZnPT**	-	17.2 ± 2.5	10.9 ± 5.4	7.6 ± 2.1	-
	**S-211N**	-	12.9 ± 1.3	11.0 ± 1.4	0.3 ± 0.0	-
	**P-A6**	-	4.1 ± 0.9	5.0 ± 1.2	2.9 ± 0.4	4.0 ± 0.3
	**CuPT**	-	2.3 ± 0.2	0.7 ± 0.0	0.5 ± 0.3	-

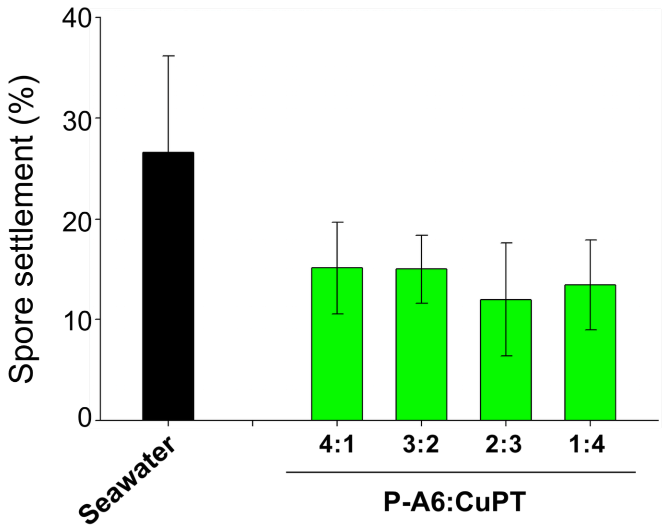

Figure S2. Settlement efficiency of *Ulva* spores in seawater containing a mixture of P-A6 and CuPT at various weight ratios at the total biocide concentration of 10 ppb.

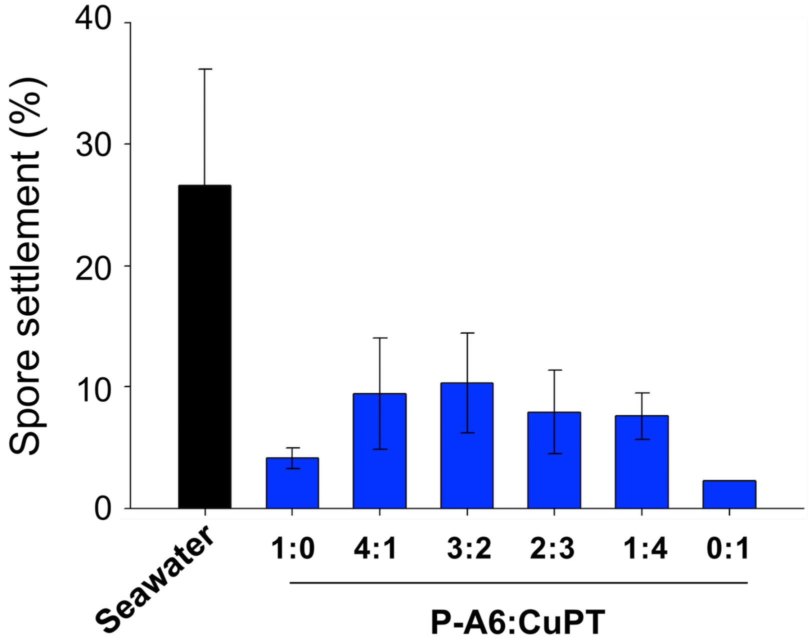

Figure S3. Settlement efficiency of *Ulva* spores in seawater containing a mixture of P-A6 and CuPT at various weight ratios at the total biocide concentration of 50 ppb.

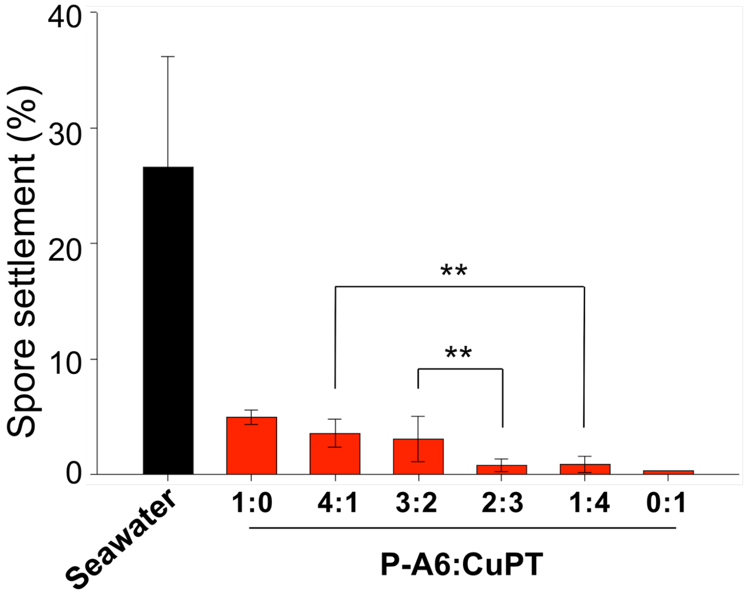

Figure S4. Settlement efficiency of *Ulva* spores in seawater containing a mixture of P-A6 and CuPT at various weight ratios at the total biocide concentration of 100 ppb. ** p-value < 0.001.

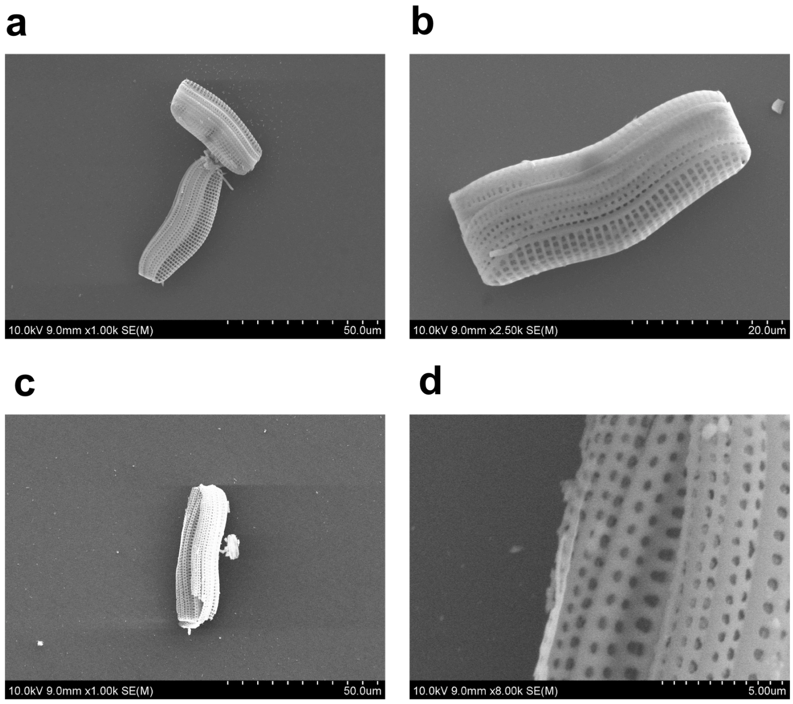

Figure S5. SEM images of Ulva spores floating in seawater after incubation under the summer condition for 24 h: seawater (a and b) and 10 ppb CuPT (c and d).

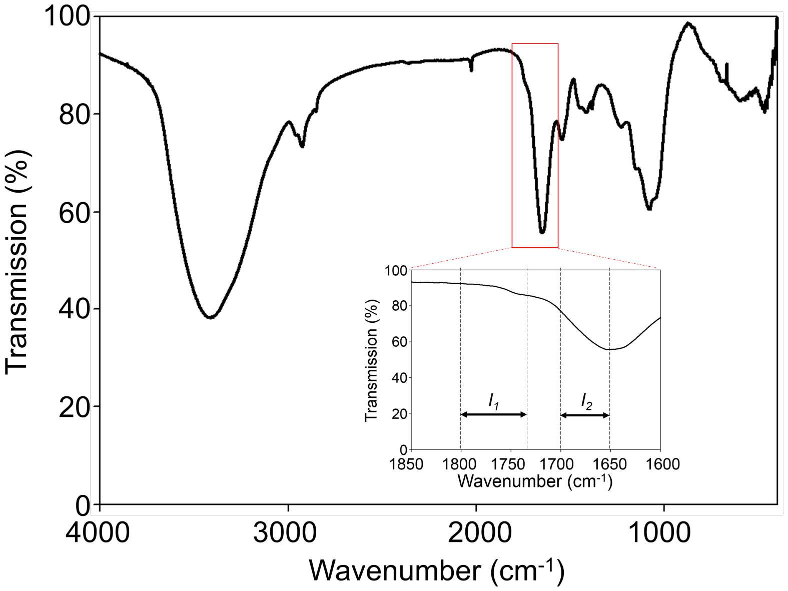

Figure S6. FTIR spectrum of adhesive pads.

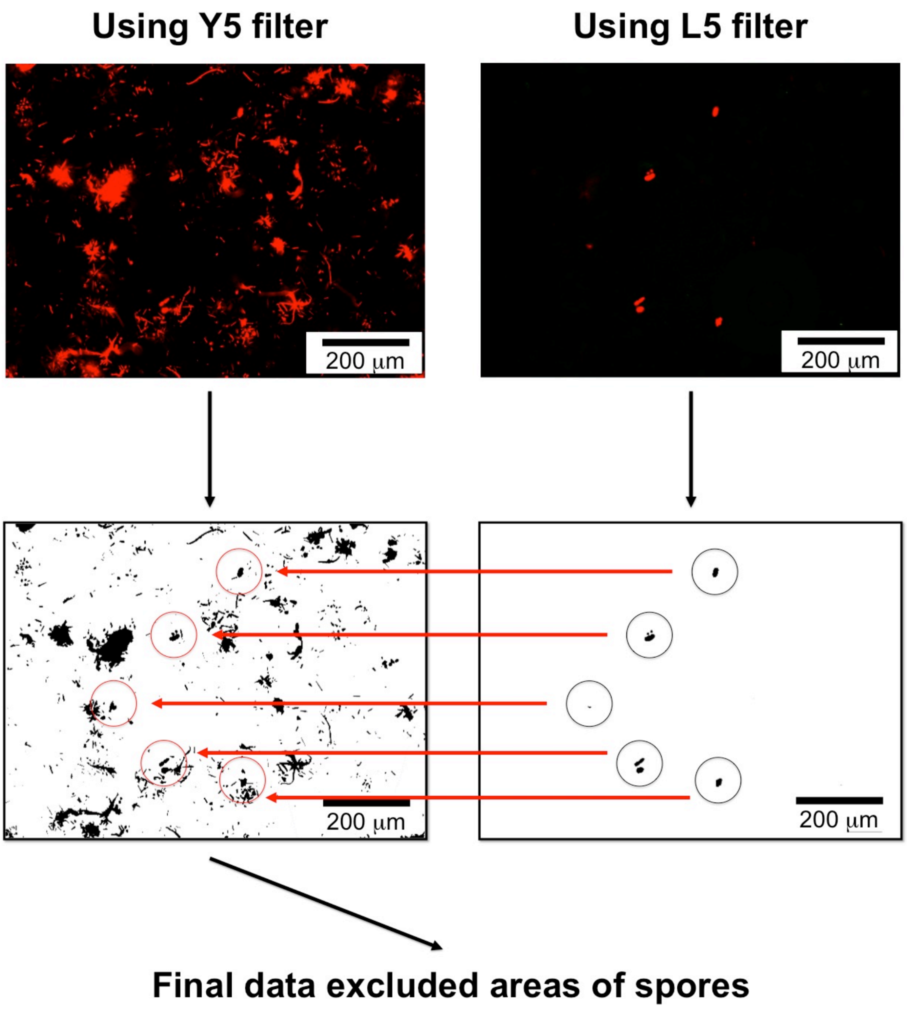

Figure S7. Fluorescence images are visualized with an Y5 filter (left) and an L5 filter (right, λ_{ex} = 480 ± 20 nm, λ_{em} = 527 ± 15 nm). Black and white images are converted from fluorescence images and analyzed using the particle analysis function in Image J. We obtained the precise locations of the spores using the L5 filter, and after subsequent image processing matched the spores positioned in the overall image taken using the Y5 filter. The area of the adhesive pads thus was quantified by excluding the areas of the matched spores.
